# Altered Salivary Flow, Protein Composition, and Rheology Following Taste and TRP Stimulation in Older Adults

**DOI:** 10.3389/fphys.2019.00652

**Published:** 2019-05-31

**Authors:** Rose-Anna Grace Pushpass, Blánaid Daly, Charles Kelly, Gordon Proctor, Guy Howard Carpenter

**Affiliations:** ^1^ Mucosal and Salivary Biology, Salivary Research, Faculty of Dental, Oral, Dental Sciences, Centre for Host Microbiome Interactions, King’s College London, London, United Kingdom; ^2^ Child and Public Dental Health, Dublin Dental University Hospital, Trinity College, Dublin, Ireland

**Keywords:** taste, saliva, Spinnbarkeit, rheology, TRP, age

## Abstract

Taste and smell perceptions diminish in older age, impacting upon quality of life and nutrition, yet the causes of taste loss are largely unknown. Transient receptor potential channels (TRP) found on the oral mucosa are also involved in oral sensations including cooling and burning and may contribute to the eating experience of older people. Older adults often have reduced salivary flow and the physical properties of saliva may change, but the role of saliva in oral sensations of older adults is yet to be elucidated. Here, the effect of older age on subjective (perception) and objective (stimulated salivary response) measures of TRP stimulants, odors, and basic tastants was investigated. Whole mouth saliva was collected from younger (mean age 24 years) and older adults (mean age 72 years) following stimulation of taste [mono sodium glutamate (MSG) and caffeine], olfaction (menthol), and TRP receptors (capsaicin). Participants rated perceived intensity of each stimulus, and salivary properties were assessed. Older age was associated with 15% lower umami taste and 26% lower menthol odor perception, coupled with 17% lower salivary response to MSG. Interestingly, there were no differences for perception of TRP stimulants, so chemo-sensation was not affected by age. Younger adults had four times greater elasticity (Spinnbarkeit) with MUC7 levels almost double and 66% greater resting salivary flow rate. Stimulated salivary responses in the younger group were also higher compared to the older group, with changes in protein and viscoelasticity in response to taste and TRP stimulation. These results show the impact of older age upon taste and smell sensation which may lead to changes in the physical and compositional properties of saliva in response to taste/odor stimulation. Measurement of stimulated salivary flow and rheology provides an objective measure of taste in addition to subjective perceptions which can be influenced by participant bias. Chemo-sensation may be retained with age and trigeminal stimuli such as chili could be employed in future studies to enhance meals for an age group at risk of malnutrition. Alteration in salivary properties due to advanced age could impact on ability to taste due to poor diffusion of tastants and reduced oral surface protection.

## Introduction

There is a link between age and the decline of taste acuity, often coupled with decreased olfactory sensation, particularly retro nasal (*via* the mouth) (Stevens and Cain, 1986). Of the five basic tastes, bitter taste may be most affected by age (Yoshinaka et al., 2016), but age related increases in taste thresholds have also been associated with salt, sour, and umami, with rate of taste loss being dependent on which substance is used in testing (Schiffman et al., 1991, 1994; Mojet et al., 2001; Jiang et al., 2016). Sweet taste is reported to be largely unaffected with age (Mojet et al., 2001; Yoshinaka et al., 2016). Sensory loss can have serious implications for the health and quality of life of an elderly individual. Their nutritional status may be impaired as a result of reduced food intake because of reduced enjoyment when eating (Schiffman, 1997). Taste loss may also result in unhealthy eating behaviors such as increased salt intake to heighten flavor in meals (Stevens et al., 1991) or eating more sugary foods (Gilmore and Murphy, 1989). Loss of sense of smell can lead to difficulty in detecting out of date foods, or failure to notice smoke or a gas leak (Boyce and Shone, 2006).

Aside from the basic tastes, other compounds in foods can elicit responses in the taste buds. Heat, pungency, cooling, astringency, and fattiness are all sensations felt on the tongue but are not considered “tastes.” Compounds such as capsaicin (chili) cause a stinging/burning sensation in the mouth due to direct activation of TRPV1 channels, expressed by epithelial cells on the tongue and oral mucosa (Marincsák et al., 2009; Wang et al., 2011). TRPM8 channels are expressed by trigeminal ganglia nerves in the tongue and in oral epithelial cells (Abe et al., 2005; Wang et al., 2011) and are activated by cold temperatures as well as by menthol and cooling agents (Peier et al., 2002; Reid et al., 2002).

Reduction in sensitivity to transient receptor potential channels (TRP) agonists could also influence the effect advanced age has on taste and hedonic aspects of eating for elderly people (Fukunaga et al., 2005). Older adults display increased sensitivity thresholds and reduced effect of repeated exposure to menthol compared to younger adults for olfaction, oral trigeminal sensation, and in foods (Murphy, 1983; Forde and Delahunty, 2002; Koskinen et al., 2003; Kremer et al., 2007). However, capsaicin sensitivity does not appear to be lost with advanced age, even in those who have loss of basic tastes (Forde and Delahunty, 2002; Fukunaga et al., 2005). Therefore, certain TRP receptor stimulants may evoke oral sensations which could increase the enjoyment of eating for older individuals who have reduced taste ability.

Saliva aids transduction of tastants by solubilizing and facilitating their movement to the taste pore where they may bind to receptor cells (Bradley et al., 2003; de Almeida, 2008). Saliva is essential to the taste response since removal of the major salivary glands results in pathological changes in the taste tissue and significant decrease in taste sensitivity (Nanda and Catalanotto, 1981; Matsuo et al., 1997). Saliva also has antibacterial and lubricating properties and provides growth factors for renewal of taste buds (Morris-Wiman et al., 2000; Mese and Matsuo, 2007).

Mucins are salivary proteins responsible for viscoelastic, muco-adhesive properties of saliva (Van der Reijden et al., 1994). There are two salivary mucins, MUC5b and MUC7, both highly glycosylated, that form non-covalent bonds to create a gel like network (Inoue et al., 2008). Surface-bound MUC1 on oral epithelial cells may facilitate muco-adhesion of the salivary pellicle by binding to salivary MUC5b (Gibbins et al., 2015; Ployon et al., 2016). This bound layer of saliva protects the oral surfaces, including the tongue, bathing the taste buds, and providing a trophic stimulus (Matsuo, 2000).

Dehydration, medication, and diseases common in older people can reduce the salivary flow, especially unstimulated whole mouth saliva (UWMS), but the effect this may have on oral sensation is unclear (Nagler and Hershkovich, 2005; Affoo et al., 2015). Parotid salivary flow is mostly retained, while submandibular/sublingual flows (responsible for 70% of resting salivary flow and 50% of stimulated) are significantly reduced (Affoo et al., 2015). As a result, protective factors such as salivary mucins, immunoglobulins, and enzymes are lower in saliva of older adults (Denny et al., 1991; Navazesh et al., 1992; Vissink et al., 1997). This may prevent lubrication of oral surfaces including the taste buds and hinder dissolution and transport of tastants to receptor cells. Few studies have investigated the effect of older age on rheology of saliva although it has been suggested that advanced age leads to increased viscoelasticity (Zussman et al., 2007). This study therefore investigated changes in multiple salivary parameters in older adults with a focus on the physical properties in order to elucidate the effects of changes in the quality of saliva on oral sensation in older adults.

In the past, taste testing has focused largely on subjective measures such as perceptions, but these are affected by individual experience and in the case of older adults, particularly, by cognitive function (Methven et al., 2016). In contrast, the salivary reflex response to oral sensory stimuli is regulated by the parasympathetic nervous system [for a review, see Proctor (2016)]. Gustatory stimuli are one of the several salivary stimuli causing increased flow rates which are concentration dependent and tastant specific (Chauncey and Shannon, 1960; Guinard et al., 1997; Neyraud et al., 2009; Ilangakoon and Carpenter, 2011). Increased labial blood flow has also been shown in response to taste stimulation and has been positively correlated to taste perceptions and labial salivary flow (Satoh-Kuriwada et al., 2018). Although saliva secretion occurs as a reflex response to taste stimuli, the increase in flow rate is not always well correlated to the perceived intensity of the tastant (Guinard et al., 1997). The absence of correlation between the two may be because every individual has different experiences with tastes, so taste perceptions are subjective, while salivary reflex response is an objective measure of taste response (Engelen et al., 2003). TRP agonists, such as capsaicin and menthol, also induce salivary secretion, above basal levels (Lawless, 1984; Nasrawi and Pangborn, 1990). The effect of TRP agonists on saliva secretion is also dependent on compound used. Also, the rheology of saliva may be altered by different TRP agonists suggesting stimulation of the mucin producing glands and parotid gland in different proportions (Houghton et al., 2017). Therefore, measurement of salivary response to taste stimulation was shown in this study as an objective method of assessing taste responses which to date has not yet been employed in sensory testing of older adults.

The aim of this study was to investigate the effect of age on subjective (self-reported perception) and objective [stimulated whole mouth saliva (SWMS) response] measures of TRP stimulants, odors, and basic tastants. UWMS and SWMS were characterized in older and younger participants for key properties which may be relevant to oral sensory sensation such as extensional rheology, pH, and protein composition. This aimed to develop an understanding of the ways older age might influence salivary characteristics and impact oral sensory sensation. The effects of different stimuli on flow rate, composition, and physical properties of saliva were also investigated to show how different taste, TRP, and odor agonists might impact characteristics of SWMS, providing an objective measure of sensory responses for use in sensory testing of older adults.

## Methods and Materials

### Study Group

Thirty-one younger (27 female, 4 males, mean age 24.3 ± 0.4 years old) and 25 older (19 female, 6 males, mean age 72.4 ± 1.8 years old) participants took part in this study. A power calculation (G*Power Software, Düsseldorf, Germany) was used to determine group size based on an average (±SD) difference in salivary flow rate and mucin concentration between older and younger adults (Navazesh et al., 1992) assuming a medium effect size (*d* = 1.12, *d* = 1.09, respectively) *n* = 20 would be required to achieve significance (*p* < 0.05) using an unpaired *t* test (95% statistical power). Smokers, pregnant or breast-feeding women and those with swallowing difficulties were excluded. Participants self-reported to be free of acute disease and infectious illnesses. This study was carried out in accordance with the recommendations of King’s College London Guidelines on Good Practice in Academic Research. The protocol was approved by the King’s College London Biomedical Sciences, Dentistry, Medicine and Natural & Mathematical Sciences research ethics committee (BDM RESC), application reference: BDM/12/13-130. All participants gave written informed consent in accordance with the Helsinki Declaration.

### Saliva Collection

Taste compounds and TRP agonists used were food grade (Sigma Aldrich, Poole, Dorset, UK) and the concentrations determined by prior testing to ensure they were above the perception threshold. Volunteers were asked to donate taste stimulated and unstimulated whole mouth saliva (UWMS) samples. A bitter tastant (caffeine) was selected to represent a basic taste with noticeable differences in sensation between age groups, while umami taste [mono sodium glutamate (MSG)] is of interest in intervention studies aimed at improving palatability of food and increasing appetite for older adults, as it does not have the negative health impact of adding salt and sugar (Schiffman et al., 1991; Schiffman, 1998; Masic and Yeomans, 2014). Capsaicin was chosen as a TRPV1 agonist and menthol as it is a TRPM8 agonist and an olfactory stimulant. Collections were carried out for 2 min for UWMS and for stimulated (SWMS) following a 1-min rinse with 1 ml taste solution [0.05 M monosodium glutamate, dissolved in spring water, 0.05 M caffeine, 0.05 M menthol, 0.005 M capsaicin, all dissolved in 1% propylene glycol (PG) and spring water]. A water rinse control was used consisting of 1% PG in spring water. For odor stimulation, a 2-min saliva collection was carried out after 1-min smelling menthol crystals. Tubes were pre-weighed and weighed again after collection of saliva to allow calculation of flow rates (g/min). Samples were stored on ice immediately after collection, aliquoted to minimize freeze thaw cycles, and stored at −80°C.

### Subjective Taste Perceptions

Participants were asked to rate the intensity of all administered tastants (including water rinse control), TRP and odor compounds using a labeled scale from no sensation to the strongest imaginable sensation [adapted from (Hayes et al., 2013)].

### Rheological Analysis

#### Extensional Rheology (ER)

The NevaMeter Extensional Rheometer (Ishikawa Iron Works, Japan) was used to carry out measurements of viscoelasticity (Spinnbarkeit) on samples. A 100 μl sample was loaded onto the machine. Measurements were conducted in triplicate and an average taken. The Spinnbarkeit was determined as the point at which electrical conductivity of the saliva was broken after being subjected to constant stretching at a rate of 5 mm/s.

### Compositional Analysis

#### Total Protein Concentration

The Bicinchoninic acid assay (BCA assay) kit (BioVision, Milpitas, USA) was used to determine the total protein concentration of samples. Bovine serum albumin (BSA) was used as a standard, 2–0.025 mg/ml. Duplicate samples of BSA at 2, 1.5, 1, 0,5, 0.25, 0.125, 0.025 mg/ml, a water control, and samples were added to a 96-well plate and absorbance at 540 nm was measured using the iMark Microplate Absorbance Reader (BIORAD, UK). Protein concentrations of samples were calculated with a standard curve created by plotting on a linear graph, the standard concentration (mg/ml) against absorbance (nm). A linear equation was generated from the graph and used to calculate sample protein amounts from absorbance readings.

#### Gel Electrophoresis

Sodium dodecyl sulfate-polyacrylamide gel electrophoresis (SDS-PAGE) was used to analyze protein composition of samples. Samples were prepared in NuPAGE lithium dodecyl sulfate sample buffer (LDS) sample buffer (25%) (Life Technologies, Paisley, UK) under reducing conditions [50 mM dithiothreitol (DTT)]. Samples were boiled for 3 min at 100°C and 50 μg total protein loaded onto the gel [Xcell electrophoresis unit (Life Technologies)] with MES SDS running buffer [50 mM MES, 50 mM Tris Base, 0.1% SDS, 1 mM EDTA, pH 7.3 (Life Technologies)] at 200 V constant for 32 min. A control sample of UWMS from one healthy donor (aged 27 years) was included in each gel to allow for normalizing between gels.

#### Periodic Acid Schiff (PAS) Glycoprotein Stain

High molecular weight glycoproteins (MUC5b and MUC7) were visualized using the PAS stain. Gels were fixed using 25% methanol, 10% acetic acid solution for 1 h. Following three, 5-min washes in Ultra High Quality (UHQ) water, gels were oxidized using 2% periodic acid solution (Sigma Aldrich). After another 2 × UHQ water washes, Schiff’s reagent was added, and gels were left to stain for 45 min in dark conditions. Water was used to de-stain, and gels were imaged using Syngene Gene Genius Bio Imaging System (Cambridge, UK).

### Normalizing Data for Analysis

When comparing between groups, taste, and TRP SWMS flow rate, Spinnbarkeit, total protein, and mucin levels were normalized to the corresponding result for water rinse control in each individual, to control for the effect of rinsing and use of carrier (PG) to dissolve some of the compounds. Olfactory SWMS results were normalized to the corresponding UWMS result. When comparing within groups, results were not normalized. The WMS ER and protein content were not significantly higher in any of the SWMS compared to UWMS (data not shown). Mouth rinsing disturbs the salivary pellicle, and as such the mucins contained within would probably be expectorated along with the mouth rinse solution. This could therefore reduce the mucin content of the WMS subsequently collected. UWMS may therefore have greater ER and mucin content. Furthermore, stimulated WMS flow rate would generally be greater than unstimulated as any stimulation, even of water alone would likely induce saliva secretion. For these reasons, data from each individual’s taste/TRP mouth rinses were analyzed as a ratio to the water rinse control (WMS), to show the effect is induced by specific stimulants and is not a result of mouth rinsing. Since the menthol odor stimulation did not involve a mouth rinse, it was appropriate to compare the results from this to unstimulated WMS. This additionally normalizes the data to each individual’s “baseline” response and thus reduces the impact of inter-individual variation.

### Statistics

GraphPad Prism 7 software (GraphPad Software Inc., La Jolla, CA) was used for statistical analysis. The data were tested for normal distribution using the D’Agostino & Pearson normality test and analyzed using non-parametric tests, Friedman test with Dunn’s multiple comparisons for significance within groups and independent Mann Whitney U test for between groups. Differences in salivary total protein were analyzed using parametric tests (one way ANOVA with Tukey *post hoc* test and student’s *t*-test) as the data were shown to be normally distributed. *Significance = *p* < 0.05 shown as * (significance between groups) and + (significance from baseline within groups).

## Results

### Subjective Taste and Odor Perceptions Were Reduced in Older Adults

**Figure 1:**
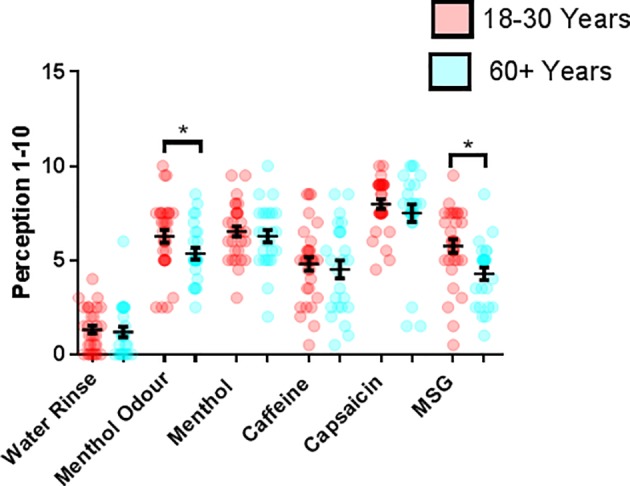
Average perceived intensity of taste and TRP agonists by subjective measurement on a scale of 0–10 in younger (18–30 years, *n* = 31) and older (60+ years, *n* = 25) participants. *p = <0.05.

To assess the effect of participant age on subjective taste, smell and TRP sensation, a labeled scale was used with a scale from 0–10, no sensation at all to strongest imaginable sensation. The average (±SEM) perception of menthol smell and the MSG mouth rinse were significantly greater in the younger participant group compared to the older 6.27 ± 0.35 and 5.36 ± 0.31 (*p* = 0.042) and 5.76 ± 0.36 (18–30) and 4.28 ± 0.34 (60+) (*p* = 0.002), respectively (Figure 1). Mouth rinses of water (control) and other taste/TRP solutions were not perceived differently between the two age groups.

### USWMS Flow Rate and SWMS Response Is Altered With Age and Is Stimulant Specific

**Figure 2:**
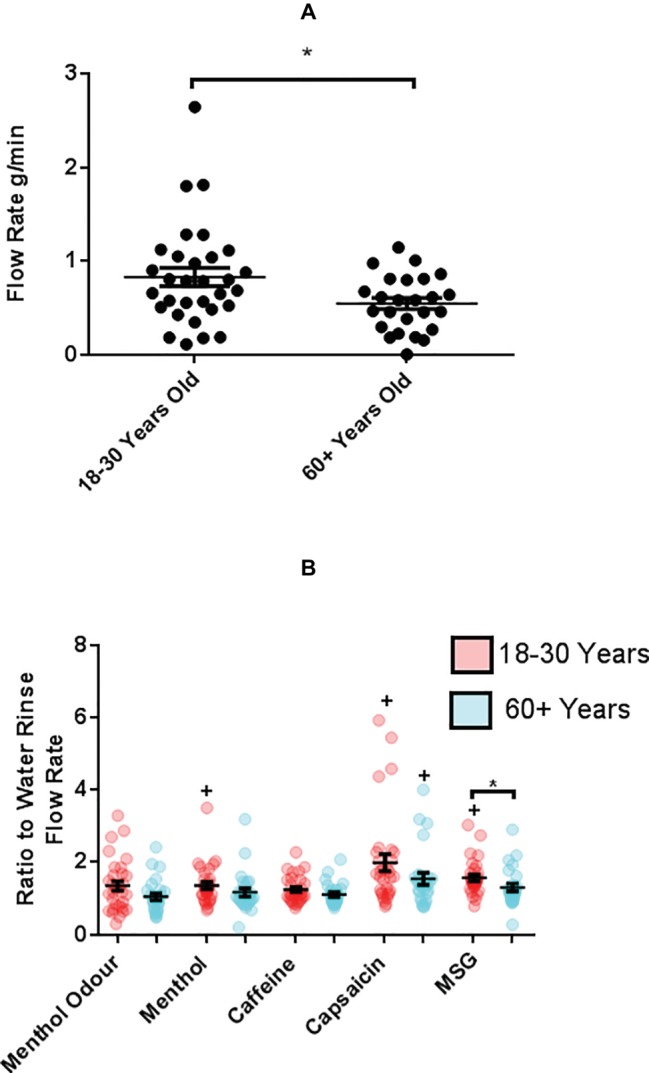
**(A)** Average (±SEM) UWMS flow rate of younger (18–30 years, *n* = 31) and older (60+ years, *n* = 25) participants. */+*p* < 0.05. **(B)** The average (±SEM) WMS flow rate following 1 min of 1 ml mouth rinse of taste and TRP agonists or 1-min smelling of menthol in younger (18–30 years, *n* = 31) and older (60+ years, *n* = 25) participants. Flow rate (g/min) following taste/TRP stimulation expressed as a ratio of flow rate (g/min) following water rinse. Flow rate (g/min) following menthol odor stimulation expressed as a ratio of resting flow rate (g/min).

The older group had a significantly lower UWMS salivary flow rate, 0.55 ± 0.06 g/min compared to 0.83 ± 0.10 g/min in the younger group (*p* = 0.044) (Figure 2A). Saliva was secreted as a reflex response to stimulus including taste and smell, and so SWMS flow rate was calculated as an objective measure of taste response. The SEM for UWMS flow rate was greater for the older group suggesting more inter-individual variation in salivary flow rates amongst this age group. When results were normalized to the water rinse stimulated flow rates (g/min), average salivary flow rate (±SEM) following mouth rinse of MSG was significantly greater in the younger group compared to the older, 1.57 ± 0.09 and 1.31 ± 0.11 (*p* = 0.009) (Figure 2B). In both age groups, there was a significant increase in salivary flow rate following mouth rinses with capsaicin compared with the water rinse control; 0.62 ± 0.05 g/min from 0.48 ± 0.05 g/min in the older (*p* = 0.013) and 2.37 ± 0.41 g/min from 1.32 ± 0.22 g/min in the younger (*p* < 0.0001). MSG mouth rinsing also induced significantly increased salivary flow in the younger group up to 2.02 ± 0.36 g/min (MSG, *p* < 0.0001). Menthol and caffeine did not evoke significantly increased WMS flow rate in either age group.

### Extensional Rheology of UWMS and SWMS Responses Were Greater in Younger Adults

**Figure 3:**
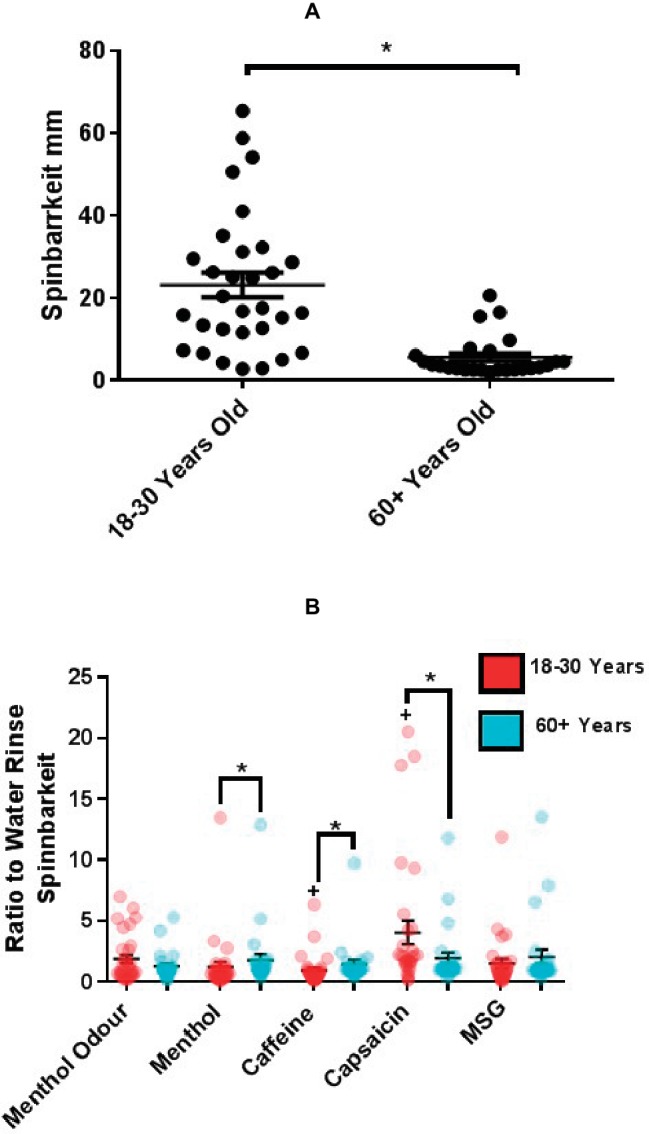
**(A)** Mean (±SEM) Spinnbarkeit (ER) of UWMS of younger (18–30 years, *n* = 31) and older (60+ years, *n* = 25) participants. */+*p* = <0.05. **(B)** Mean (±SEM) Spinnbarkeit (ER) of WMS following 1 min of 1 ml mouth rinse of taste or TRP agonist or 1-min smelling of menthol of younger (18–30 years, *n* = 31) and older (60+ years, *n* = 25) participants, assessed using the NevaMeter. ER (mm) following taste/TRP stimulation expressed as a ratio of ER (mm) following water rinse. ER (mm) following menthol odor stimulation expressed as a ratio of resting ER (mm).

Extensional rheology (ER) or Spinnbarkeit gives a measure of the stringiness of saliva, which may be important for transduction of tastants to the taste pore. To investigate whether the physical properties of saliva were altered with age following taste, smell, and TRP stimulation, all saliva samples were measured using the NevaMeter for ER. As shown in Figure 3A, the average (±SEM) ER of UWMS was significantly greater in the group of younger adults compared to older, 23.09 ± 3.02 mm (18–30) and 5.59 ± 0.99 mm (60+) (*p* < 0.0001), indicating an effect of age not only on the quantity of saliva produced but also its physical properties. Menthol and caffeine mouth rinsing induced significantly lower ER in the younger group, 1.23 ± 0.43 (menthol) and 0.94 ± 0.22 (caffeine) compared to 1.78 ± 0.50 (menthol) and 1.44 ± 0.36 (caffeine) in the older group, when using ER of WMS following water rinse as a baseline (*p* = 0.002 and *p* = 0.0008, respectively, Figure 3B). Capsaicin evoked the highest increase in elasticity in the younger group, significantly greater than baseline, up to 35.26 ± 5.24 mm from 17.29 ± 3.42 mm (*p* = 0.002), as well as significantly greater than the older group, 4.05 ± 0.98 compared to 1.94 ± 0.49 ratio to water rinse baseline (*p* = 0.009, Figure 3B). Neither rinsing with MSG nor smelling of menthol had any significant effect on the ER of WMS (Figure 3B).

### Age and Stimulation Alter Salivary Total Protein Levels and Unstimulated Salivary MUC7 Is Reduced in Older Adults

**Figure 4:**
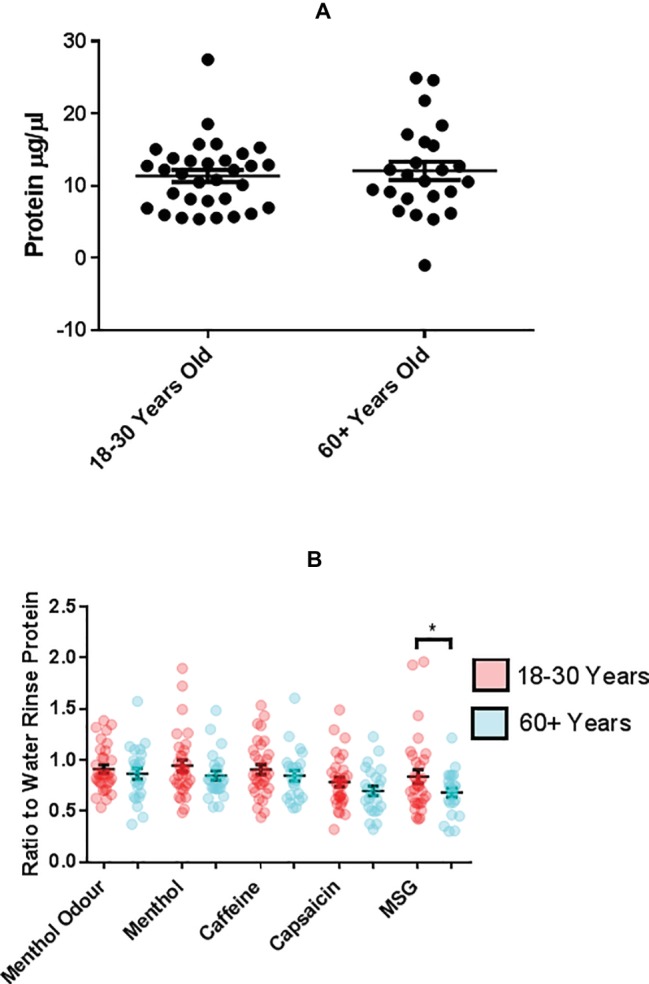
**(A)** Mean (±SEM) total protein of UWMS of younger (18–30 years, *n* = 31) and older (60+ years, *n* = 25) participants. *p = <0.05. **(B)** Mean (±SEM) total protein of WMS following 1 min of 1 ml mouth rinse of taste or TRP agonist or 1-min smelling of menthol of younger (18–30 years, *n* = 31) and older (60+ years, *n* = 25) participants, assessed using BCA assay. Total protein (μg/μl) following taste/TRP stimulation expressed as a ratio of total protein (μg/μl) following water rinse. Total protein (μg/μl) following menthol odor stimulation expressed as a ratio of resting total protein (μg/μl).

Salivary proteins, especially mucins, contribute to the viscoelastic properties of saliva. Since the flow rate and ER of saliva were altered by different stimuli and appear to be reduced with advanced age, the protein composition of saliva samples was investigated. The average (±SEM) total protein content of UWMS was not significantly different between the age groups, 11.35 ± 0.83 μg/μl (18–30 years) and 12.04 ± 1.27 μg/μl (over 60 years) (Figure 4A). The SEM for total protein (μg/μl) in unstimulated WMS was greater for the older group suggesting a greater degree of heterogeneity for protein levels in saliva amongst this age group. MSG mouth rinsing induced WMS with significantly higher protein levels in the younger group compared to the older, 0.84 ± 0.07 (arbitrary values, ratio to water rinse WMS protein) (18–30 years) and 0.68 ± 0.04 (*p* = 0.0164, arbitrary values, ratio to water rinse WMS protein) (60+ years) (Figure 4B). There was no significant difference between protein levels in WMS while smelling of menthol or following mouth rinses with menthol, caffeine, and capsaicin between the two age groups (Figure 4B). Additionally, none of the mouth rinses or smelling of menthol induced WMS with altered total protein level from baseline (water mouth rinse or resting saliva, respectively) (Figure 4B).

### Reduced MUC7, but Not MUC5b, in the Older Group Compared to the Younger Group

**Figure 5:**
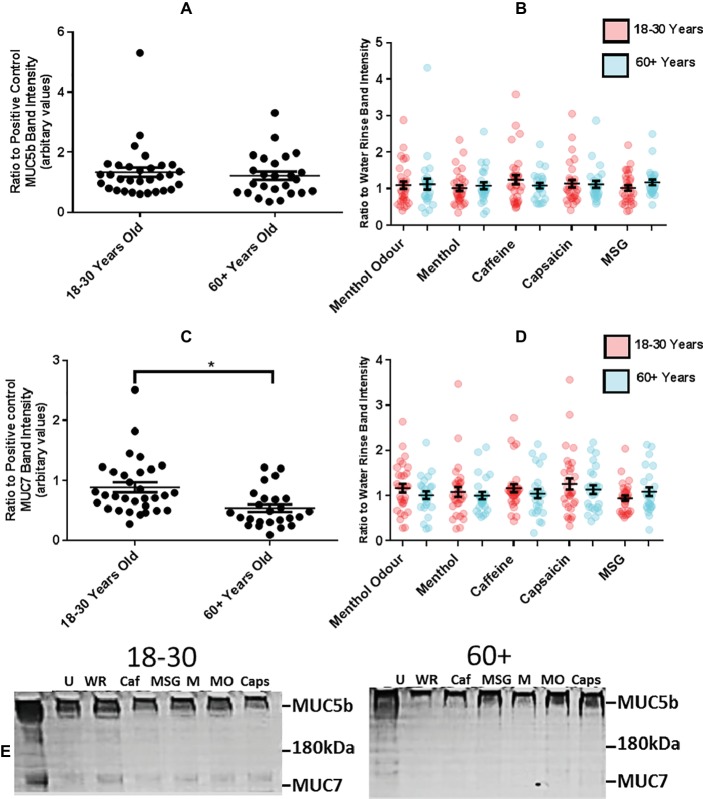
**(A)** Mean (±SEM) MUC5b level in UWMS of younger (18–30 years, *n* = 31) and older (60+ years, *n* = 25) participants. **(B)** Mean (±SEM) MUC5b level in stimulated WMS following mouth rinse of taste or TRP agonist or smelling of menthol in younger (18–30 years, *n* = 31) and older (60+ years, *n* = 25) participants (band intensity on a PAS stained SDS-PAGE gel expressed as a ratio of water rinse/unstimulated MUC5b band intensity). **(C)** Mean (±SEM) MUC7 level in UWMS of younger (18–30 years, *n* = 31) and older (60+ years, *n* = 25) participants (*p = <0.05). **(D)** Mean (±SEM) MUC7 level in stimulated WMS following mouth rinse of taste or TRP agonist or smelling of menthol in younger (18–30 years, *n* = 31) and older (60+ years, *n* = 25) participants (calculated as band intensity on a PAS stained SDS-PAGE gel expressed as a ratio of water rinse/unstimulated MUC7 band intensity). **(E)** Representative image of an SDS-PAGE gel PAS stained for mucin glycoproteins. U, unstimulated; WR, water rinse; Caf, caffeine; MSG, mono sodium glutamate; M, menthol; MO, menthol odor; Caps, capsaicin. Full gel images shown in Supplementary Figures 1, 2.

Since extensional rheology but not total protein in UWMS was affected by age, further analyses of individual proteins were carried out, to see whether relative proportions were altered. Salivary mucins, especially MUC7, are largely responsible for the ER of WMS. As the ER was different between age groups and dependent on stimulus, the relative levels of mucins, MUC5b and MUC7, were semi quantified using PAS staining of SDS-PAGE gels (Figure 5E) to see if age or stimulant caused secretion of altered levels of mucin which could explain the altered ER. The average level of MUC5b in USWMS or SWMS was not different between age groups (Figure 5A); however, there were significantly higher amounts of MUC7 in the UWMS of the younger group compared to the older, 0.89 ± 0.08 compared to 0.54 ± 0.06 (arbitrary units, *p* = 0.0004) which may explain the higher ER of younger resting WMS (Figure 5C). None of the taste or TRP mouth rinses or the menthol smelling evoked WMS with MUC5b levels that differed significantly from baseline (Figure 5B). However, the MUC7 level in capsaicin stimulated saliva from the younger group was the highest compared to baseline out of all the stimulated WMS tested, on average 26.1% (±12.51%) greater (not statistically significant) (Figure 5D). As such, the increased ER in capsaicin stimulated WMS in the younger group may partly be due to increased secretion of MUC7.

## Discussion

While taste perceptions are subjective and rely on the individual’s experiences and environmental factors, salivary flow occurs as an autonomic reflex response to taste stimulation and is an objective measure. There was some correlation between taste and smell perception and salivary flow rate as reduced umami taste perception was coupled with a reduced salivary flow rate in the older group. Capsaicin significantly increased salivary flow compared to water in both age groups, more so in the younger, indicative of greater sensation in the younger participants. Although not statistically significant, there was generally a greater salivary response to all the stimulants in the younger group, even when perceptions were similar between groups, such as for menthol and caffeine. SWMS flow rate may be a more sensitive, objective measure of taste ability than subjective reporting. This method of assessment could prove especially useful in older adults who may have a decline in cognitive function which might otherwise affect accuracy of results from sensory testing. Only one concentration of each stimulant was tested to allow for testing of a greater range of stimuli without adding to the time burden on participants, but more differences may have been seen for taste, odor, and TRP perceptions and responses if a variety of concentrations had been used.

The results in this study may be underestimating the effect of older age on taste loss in the population since women may experience taste loss with reduced severity compared to men and there were more female participants in both age groups. Several studies have demonstrated that there is no effect of gender on taste function in young participants, but men are more susceptible to age related decline in taste acuity (Yamauchi et al., 2002; Wardwell et al., 2009; Heft and Robinson, 2010; Yoshinaka et al., 2016).

Older age was also associated with reduced menthol odor perception in this study. Previous studies also reported lower perceptions in older adults for olfaction, in particular for menthol odor perception (Murphy, 1983). Interestingly, there were no differences for perception of oral TRP stimulants, unlike basic taste and smell, so chemo-sensation was not impaired in older age. It was expected that capsaicin sensation would be retained in the older age group as this has been shown previously (Forde and Delahunty, 2002; Fukunaga et al., 2005). However, it has previously been suggested that perceptions of menthol solution administered orally are reduced in older adults (Koskinen et al., 2003; Kremer et al., 2007). Discrepancies in findings may be due to differences in the testing method employed. It is interesting that menthol odor perception was reduced in the older group, while perception of the mouth rinse, which would be perceived by a combination of trigeminal stimulation and retro nasal olfaction, was not reduced. This finding indicates that it is loss of olfactory function and not trigeminal sensation that leads to reduced perception of menthol in older adults. Certain TRP agonists may therefore be useful in improving hedonic aspects of eating and encouraging food intake in older adults with taste loss, as their sensation is not impaired by advanced age.

Even though perception of oral TRP stimuli appeared to be retained with advanced age, the salivary reflex responses to menthol and capsaicin differed between the age groups. Perceptions of TRP stimuli may be affected by the different experiences and environment of older adults, while salivary responses are not. It may be that the older generation is less used to or accepting of different sensations in food because certain ingredients were not commonly available until more recently. It is known that childhood eating experiences play an important role in attitudes and behaviors later in life (Edfors and Westergren, 2012). This could apply to chili or capsaicin as well as menthol (derived from mint) as imported herbs and spices are examples of foods, which were not readily available due to rationing from 1940 to 1954 in the UK (Foster and Lunn, 2007). Previous studies have found that liking of spicy foods is reduced in older adults (Guido et al., 2016). Older people may therefore report that the sensation is stronger than is being sensed physiologically because they are less accepting of oral burning or cooling. Conversely, SWMS reflex response may be impaired in older adults. Age-related reduction in SWMS flow can be linked to an overall reduction in submandibular/sublingual flow. However, age-related reduction in UWMS flow is around 66% greater than for stimulated flow, suggesting that the reflex salivary response is somewhat retained compared to resting salivary flow (Affoo et al., 2015). Altered resting salivary composition could also play a role in reduced response to TRP agonists, since if the quality of older people’s saliva is reduced, it may transduce the stimulants to the receptors less well than in a younger individual. As such, there may be altered TRP receptor activation in older age and salivary response is therefore impaired following stimulation.

Since saliva is required for normal taste function, reduced flow rate and altered physical properties might play a role in impaired taste sensation. Saliva with high viscoelasticity is likely to be highly muco-adhesive and therefore provide improved surface wetting and protection of oral surfaces including the tongue. Additionally, saliva is a solvent in which tastants are solubilized to be transported to taste receptors. In the present study, we demonstrated that flow rate and viscoelasticity of saliva may reduce with age as ER and flow of resting saliva was significantly greater in the younger participants compared with older. It could be hypothesized therefore that older adults with reduced salivary ER might also have reduced taste sensation because of poor muco-adhesion of saliva on the oral epithelium which may mean saliva cannot transduce or solubilize tastants as well. Additionally, a reduced flow rate could also affect taste function since less saliva present in the mouth may mean oral surfaces less well protected by the salivary layer, perhaps leading to atrophy of taste cells. These findings contrast with the results shown by Zussman et al. suggesting that saliva from older people had higher viscoelasticity (Zussman et al., 2007). This may be due to differences in testing conditions as the latter study used an elongational viscometer and measured relaxation times, while in the present study, extensional viscoelasticity was measured as Spinnbarkeit or stringiness.

Previous studies measuring Spinnbarkeit using the NevaMeter method have shown ER of healthy UWMS to be anywhere from 44.0 ± 5.9 to 28.5±5.7 mm, while for xerostomic patients, this value drops to 9.2±4.3 mm, (Chaudhury et al., 2015; Houghton et al., 2017). This suggests that the younger group was within the range of “healthy,” while the older group had similar ER range to xerostomic patients. This is probably due to the submandibular/sublingual salivary flow being subject to the greatest age-related reduction, while parotid salivary flow, which does not contain mucin, is unaffected by age suggesting that the saliva of an older individual contains a greater proportion of parotid saliva (Affoo et al., 2015). It is known that parotid saliva has no mucin content and therefore also has a significantly lower viscoelasticity compared to submandibular/sublingual saliva (Zussman et al., 2007; Vijay et al., 2015). Previously, it has been shown that Spinnbarkeit can be a sensitive measure for identifying Sjögren’s syndrome as it is reduced even in cases where the patient suffering dry mouth has a normal flow rate (Chaudhury et al., 2016). Along with the results presented here, this shows that Spinnbarkeit can be an important measure of the “quality” of saliva which may have equal importance as flow or “quantity” of saliva.

In a recent study from our group (Houghton et al., 2017), it was hypothesized that TRP stimulants may be useful in relieving dry mouth in xerostomic patients. The present results support the idea that TRP agonists, particularly capsaicin, could stimulate secretion of WMS with greater muco-adhesion and viscoelasticity which might reduce feelings of dry mouth. However, this may only be useful in young dry mouth patients, as neither of the TRP agonists tested here caused saliva secretion with increased ER in the older participants. Houghton et al. (2017) also demonstrated that menthol evoked WMS had increased ER, which was not the case here. The difference in results may be because the previous study had only six participants, therefore inter-individual variation could have greater effect on the results. Additionally, WMS was collected in the first minute after stimulation separately from the second minute. Any increase in ER following menthol stimulation was only seen during the first minute and therefore was said to be an instant and transient effect. It was suggested that mucin stores could have been depleted following the initial stimulation of saliva secretion, so that WMS expectorated subsequently had lower ER. In the present study, saliva was collected for 2 min after stimulation and the initial ER stimulating effect of menthol may have been partially masked.

Salivary mucin can be correlated to ER as mucins, especially MUC7, form a viscoelastic gel like network *via* non-covalent bonding (Inoue et al., 2008). Thus, significantly more MUC7 in the younger group could generate significantly greater ER compared to the older group. There were no significant differences in mucin levels in SWMS in either group; however, a trend was apparent since capsaicin rinsing, which evoked a highly viscoelastic saliva in the younger group, also evoked saliva with the greatest levels of MUC7 compared to water rinse and all other rinses. Lack of significant difference in mucin levels in SWMS may be due to high inter-individual variation in salivary composition. This might also be reflective of high inter-individual variation in responses to stimulation as altered composition may be a direct result of SWMS secretion in varying proportions from the different saliva glands.

Generally, there were high levels of inter-individual variation especially amongst the older group for salivary flow rate, protein levels, and mucin amounts. This highlights the lack of homogeneity in salivary properties amongst the older population. This high level of inter-individual variation has been highlighted in previous studies and as such, heterogeneity in salivary properties following taste, TRP or smell stimulation could be due to high inter-individual variation in sensation in older adults (Boesveldt et al., 2018). Outliers in the younger group were seen in the case of total protein in MSG SWMS and capsaicin SWMS flow rate, which led to a greater SEM. This may be caused by marginal subsets of individuals with particularly high or low sensitivity to capsaicin or MSG, perhaps due to differences in dietary consumption of these compounds, and thus a reduced or increased salivary response.

As mentioned above, there was a larger proportion of female participants in this study, and women generally have lower salivary flow rates than men (Percival et al., 1994). Yet, the age-related decline in salivary flow is proportional between genders (Affoo et al., 2015). Hormones such as estrogen can influence salivary composition, and females have smaller salivary glands that may affect saliva secretion and composition (de Almeida et al., 2008). Females also have lower salivary pH and reduced buffering capacity, total protein, MUC5b, and secretory IgA levels but increased MUC7 and lysozyme activity compared to males (Prodan et al., 2015). Also, the differences in taste and salivary parameters between older and younger adults observed in the present study are likely not a result of age alone and may result from multiple factors occurring as a consequence of the ageing process such as presence of diseases, intake of medications, and differences in lifestyle.

## Concluding Remarks

Taken together, these results show the impact of advanced age upon taste and smell sensation, while trigeminal sensations may be less affected by age. These changes in sensation may also lead to reduced salivary response to taste, TRP, and odor stimulation in older adults. This is because these stimuli can cause altered physical and compositional properties of saliva as a reflex response in young healthy adults. As such, measurement of salivary flow rate and even extensional rheology could be a useful tool for sensory testing as it is not affected by individual experience and cognitive factors which can impact upon sensory perceptions. Salivary flow and elasticity were reduced in older adults, and this may play a role in impaired taste function. Future work could be directed toward development of artificial saliva with high viscoelasticity and muco-adhesive properties that could be used to improve taste function in the older population.

## Ethics Statement

This study was carried out in accordance with the recommendations of King’s College London Guidelines on Good Practice in Academic Research. The protocol was approved by the King’s College London Biomedical Sciences, Dentistry, Medicine and Natural & Mathematical Sciences research ethics committee (BDM RESC), application reference: BDM/12/13-130. All subjects gave written informed consent in accordance with the Helsinki Declaration.

## Author Contributions

R-AP designed the experiments in discussion with GC and CK. R-AP performed the experiments and analyzed the data. BD assisted with eligibility screening of participants by performing oral health checks prior to the testing. R-AP wrote the manuscript in consultation with CK, GP, and GC.

### Conflict of Interest Statement

The authors declare that the research was conducted in the absence of any commercial or financial relationships that could be construed as a potential conflict of interest.

## Abbreviations

ATP: Adenosine triphosphate
BCA: Bicinchoninic acid
CBB: Coomassie Brilliant Blue R250
ER: Extensional rheology
MSG: Monosodium glutamate
PAS: Periodic acid-Schiff’s stain
SDS-PAGE: Sodium dodecyl sulfate-polyacrylamide gel electrophoresis
SWMS: Stimulated whole mouth saliva
T1R: Taste receptor type 1
TRP: Transient receptor potential channel
UWMS: Unstimulated whole mouth saliva
WMS: Whole mouth saliva.
