# Supplementary figures and images for: Altered Salivary Flow, Protein Composition, and Rheology Following Taste and TRP Stimulation in Older Adults
Source: Front Physiol. 2019 May 31;10:652. doi: 10.3389/fphys.2019.00652 (PMC6555201; doi:10.3389/fphys.2019.00652)

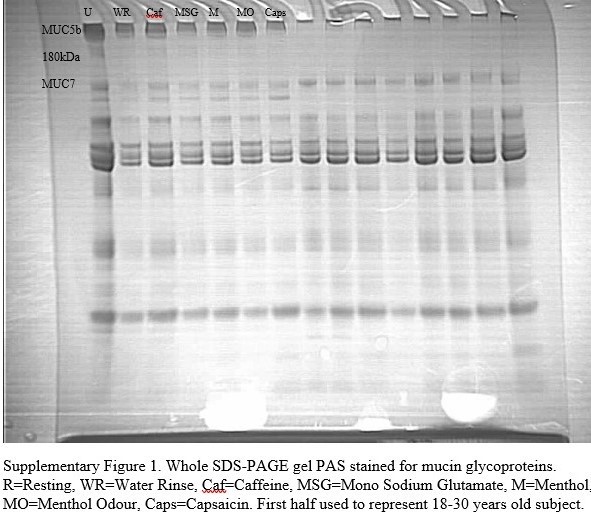

Supplement: Supplementary file 1 [file image_1.jpeg]

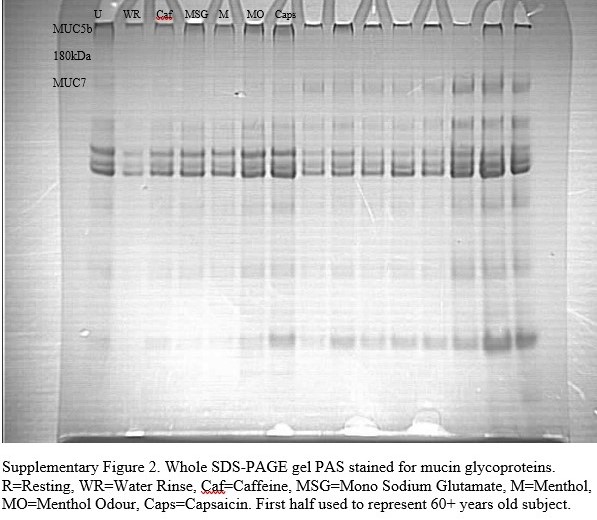

Supplement: Supplementary file 2 [file image_2.jpeg]
